# Honey bees with a drinking problem: potential routes of *Nosema ceranae* spore transmission

**DOI:** 10.1017/S0031182021001827

**Published:** 2022-04

**Authors:** Courtney I. MacInnis, B. Andrew Keddie, Stephen F. Pernal

**Affiliations:** 1 Department of Biological Sciences, University of Alberta, Edmonton, Alberta, Canada T6G 2E9; 2 Agriculture and Agri-Food Canada, Beaverlodge Research Farm, P.O. Box 29, Beaverlodge, Alberta, Canada T0H 0C0

**Keywords:** *Apis mellifera*, infectivity, *Nosema ceranae*, spore, viability

## Abstract

*Nosema apis* and *N. ceranae* are the two causative agents of Nosema disease in adult honey bees (*Apis mellifera* L.). *Nosema apis* has been a recognized parasite for over a century and its epizootiology is well known. In contrast, *N. ceranae* is an emerging parasite of honey bees, which is now globally prevalent and the dominant *Nosema* spp. in many parts of the world. Despite this, many gaps in our knowledge exist regarding this species. For example, we do not fully understand all of the routes of transmission of *N. ceranae* among bees, or how long this parasite is capable of surviving in honey bee colonies. Here we investigated the viability and infectivity of *N. ceranae* spores in water and 2 M sucrose over time after storage at 33, 20, −12 and −20°C. Spores in both 2 M sucrose and water maintained high viability, except in water at −20°C over the course of the 6-week experiment. Infectivity was variable for spores after storage at all four temperatures, but all were infective at the last time point. The results provide evidence for cold tolerance and suggest that both water and 2 M sucrose (fall bee feed) could act as routes of transmission for *N. ceranae*. This work also contains information that may help influence management recommendations for the parasite.

## Graphical abstract

**Figure:**
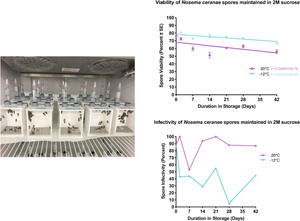

## Introduction

*Nosema apis* Zander and *N. ceranae* Fries *et al*., 1996, recently proposed to be reclassified as *Vairimorpha ceranae* (Tokarev *et al*., 2020), are the two causative agents of Nosema disease in adult honey bees (*Apis mellifera* L.). As *N. apis* has been a known parasite of honey bees for over 100 years (Zander, 1909), its epizootiology is well documented (see Fries, 1997). Less is known about details of the life cycle of *N. ceranae*, as it was only described from honey bees in the early 2000s (Higes *et al*., 2006; Huang *et al*., 2007). Despite its more recent discovery, this parasite now has a nearly global distribution and a number of alternative hosts (Holt and Grozinger, 2016; Goblirsch, 2018).

What is known about *N. ceranae*'s epizootiology, particularly in individual honey bees, is that the parasite is rather insidious. *Nosema ceranae* degenerates midgut tissues (Dussaubat *et al*., 2012; Panek *et al*., 2018), suppresses the immune system (Antúnez *et al*., 2009), causes iron deficiencies (Rodríguez-García *et al*., 2021) and decreases the lifespan and nursing ability of adult bees (Higes *et al*., 2007; Goblirsch *et al*., 2013). *Nosema ceranae* is also capable of impairing olfactory learning (Gage *et al*., 2018) and altering flight behaviour (Dussaubat *et al*., 2013), causing energetic stress (Mayack and Naug, 2009; Li *et al*., 2018), and may induce precocious foraging (Mayack and Naug, 2009; Goblirsch *et al*., 2013; Li *et al*., 2019). At the colony level, the effects of *N. ceranae* are more variable. Studies in northern Europe show no clear relationship between *N. ceranae* infection and colony mortality (Genersch *et al*., 2010; Gisder *et al*., 2010). In contrast, studies in Spain have shown that the parasite is capable of reducing brood-rearing capacity and honey production leading to the eventual collapse of some colonies (Higes *et al*., 2008, 2009; Botías *et al*., 2013), though the parasite may also be found in colonies not exhibiting obvious colony-level symptoms (Fernández *et al*., 2012). In the USA, metagenomic analyses have shown that *Nosema* spp. co-infections were one of few pathogenic metrics that differentiated CCD-affected colonies from healthy colonies (Cox-Foster *et al*., 2007; vanEngelsdorp *et al*., 2009). Additionally, Canadian beekeepers have frequently cited *Nosema* spp. infections as a leading cause of colony mortality (Claing *et al*., 2020).

Several studies have attempted to identify treatment alternatives to Fumagilin-B^®^ and Fumidil-B^®^ for suppressing *N. ceranae* (see Holt and Grozinger, 2016), however these fumagillin-based treatments remain the only registered chemotherapeutic therapies against the parasite (Williams *et al*., 2008, 2011; Higes *et al*., 2011; van den Heever *et al*., 2015*a*). Unfortunately, fumagillin is only effective against the replicating stages of the parasite, not the spore stage, making it difficult to eliminate active infections. Fumagillin was originally developed as a treatment for *N. apis* infections in North America, over 60 years ago (Katznelson and Jamieson, 1952; Bailey, 1953). Since that time, evidence suggests that fumagillin may actually exacerbate *N. ceranae* infections at low concentrations (Huang *et al*., 2013), and causes some toxicity to adult honey bees in its commercial formulation as a dicyclohexylamine salt (van den Heever *et al*., 2015*b*). As *N. ceranae* is an emerging parasite of the honey bee exhibiting few visible symptoms (Fries *et al*., 2006; Higes *et al*., 2008; Stevanovic *et al*., 2013; Horchler *et al*., 2019), the routes of transmission among and within colonies remain poorly understood. This gap in knowledge also makes the development of effective alternative management strategies more challenging.

Several studies have evaluated the thermotolerance of *N. ceranae* spores using laboratory assays as well as in honey bees. Most reported that the parasite is thermotolerant but sensitive to low temperatures (Fenoy *et al*., 2009; Fries and Forsgren, 2009; Martín-Hernández *et al*., 2009; Gisder *et al*., 2010, 2017; Higes *et al*., 2010; Sánchez Collado *et al*., 2014), while one has shown that the parasite is capable of surviving cryogenic storage conditions (McGowan *et al*., 2016). This narrative is confusing given that *N. ceranae* is able to survive and proliferate in both temperate and warmer climates, putatively displacing *N. apis* in many regions (Chauzat *et al*., 2007; Paxton *et al*., 2007; Williams *et al*., 2008; Invernizzi *et al*., 2009; Tapaszti *et al*., 2009; Stevanovic *et al*., 2011; Traver and Fell, 2011; Martín-Hernández *et al*., 2012; Emsen *et al*., 2016), but not others (Gisder *et al*., 2010, 2017). MacInnis *et al*. (2020) evaluated the survival of *N. ceranae* spores in substrates associated with honey bee colonies, and found evidence for temperature-dependent survival in some substrates, notably with spores stored in honey surviving up to one year at −20°C. This finding illustrated that *N. ceranae* spores may be transmitted through honey, and that the presence of *N. ceranae* in temperate regions is therefore not enigmatic. Here, our goal was to evaluate the viability (ability to survive) and infectivity (ability to generate infection) of *N. ceranae* spores in two liquids commonly associated with beekeeping, in an effort to further understand how this parasite survives and how it may be transmitted within and among colonies. We evaluated *N. ceranae* spore viability and infectivity in water, a liquid all colonies require, and 2 M sucrose, which beekeepers feed colonies in temperate climates in preparation for winter. This work will add to our existing knowledge of spore biology and provide for the development of strategies that do not rely on antimicrobials, so as to reduce the spread of the parasite and improve honey bee health.

## Materials and methods

Honey bee colonies managed by Agriculture and Agri-Food Canada's (AAFC) Apiculture Program at Beaverlodge Research Farm (55°11′43.0″N; 119°17′57.3″W), and a cooperating beekeeper in Girouxville, Alberta, were sampled for the presence of *N. ceranae* in May 2015. Samples of 60 worker bees were collected from each colony and processed for spore counts and *Nosema* spp. identification. Samples confirmed to be infected with only *N. ceranae* were used to infect newly-eclosed *Nosema* spp.-free bees, in order to propagate, harvest and purify the large number of spores required for experiments. For methodological details regarding these processes, the reader is directed to MacInnis *et al*. (2020).

### Experimental design, inoculation of liquids and recovery of spores

The viability and *in vivo* infectivity of the *N. ceranae* spores were evaluated after storage at one of four temperature treatments (33, 20, −12 and −20°C) at several time intervals: 7, 9, 14, 21, 28 and 46 days after storage for water, and 2, 7, 14, 21, 28 and 42 days after storage for 2 M sucrose. *Nosema ceranae* spore viability was evaluated using fluorescent microscopy at each of the time intervals, while infectivity was assessed by inoculating newly-emerged bees with the temperature-treated *N. ceranae* spores after their time in storage at the aforementioned time intervals. These bees were then evaluated 14 days later to determine if the temperature-treated spores were infective. The temperature treatments were chosen to reflect the broodnest temperature of a colony (33°C), a room temperature control (20°C) and typical fall (−12°C) and winter (−20°C) temperatures that colonies experience in northern temperate climates. Intervals were also chosen to be comparable with other *N. ceranae* viability studies (see Goblirsch, 2018). Temperature treatments at 33 and 20 ± 1.0°C were maintained in programmable incubators (models I36NLC8, I36NLC9, Percival Scientific, Perry, IA, USA) while −12 and −20 ± 1.5°C treatments were maintained in programmable freezers. Temperature profiles over time were monitored with dataloggers (Hobo TidbiT v2 Temp Logger, Cape Cod, MA, USA).

#### Water

A purified *N. ceranae* spore pellet (containing ~3.6 × 10^8^ spores) was resuspended in 2 mL of Type I sterile water and brought to a final volume of 2.89 mL [see MacInnis *et al*. (2020) for details regarding spore pellet generation]. This suspension was thoroughly mixed to ensure equal distribution of spores, and then used to create 180 500 *μ*L aliquots (~2.0 × 10^6^ spores per aliquot). These aliquots were placed in sterile 1.5 mL microcentrifuge tubes, and then evenly distributed among the four temperature treatments, such that 45 500 *μ*L aliquots were exposed to each temperature treatment.

The number of spores per mL was estimated for these water aliquots after specified time intervals according to Cantwell (1970) using a Helber Z30000 counting chamber. This was done in an effort to prepare the required number of spores for viability assays using fluorescent microscopy, and *in vivo* infectivity assays.

#### Sucrose

*Nosema ceranae* spores (~7.3 × 10^8^) were suspended in 500 *μ*L of Type I sterile water and were added to 80 mL of 2 M sucrose (sucrose + sterile Type I water as solvent). This 2 M sucrose/spore suspension was swirled continuously in a flask to ensure even spore suspension while creating 160 500 *μ*L aliquots (~4.6 × 10^6^ spores per aliquot). These aliquots were stored in 1.5 mL microcentrifuge tubes and divided evenly among the four temperature treatments, such that 40 500 *μ*L aliquots were exposed to each temperature treatment.

To recover spores, 1 mL of water was added to each aliquot, and mixed to ensure homogenization. Number of spores per mL was then estimated as described above, and aliquots prepared for viability and *in vivo* infectivity assays. We chose to evaluate the viability and infectivity of *N. ceranae* spores in 2 M sucrose as this concentration mimics that of the sucrose given to colonies by beekeepers in the fall to help prepare the colonies for winter in northern temperate climates.

### Viability

Viability was assessed using the fluorescent stains 4′,6-diamidino-2-phenylindole (DAPI) dilactate, and propidium iodide (PI). DAPI is a nucleic acid counterstain, whereas PI is a viability stain (Molecular Probes, 2006*a*, 2006*b*). These stains were chosen as they do not overlap in excitation or emission spectra, allowing living and dead spores to be reliably differentiated based on colour; working stocks were prepared per MacInnis *et al*. (2020). Viability stains were added simultaneously to aliquots destined for viability assessments (2 *μ*L of each dye per 2.0 × 10^6^ spores in 20 *μ*L water), and incubated at room temperature in the dark for 20 min.

After incubation, aliquots were centrifuged at 800 ***g*** for 6 min. The supernatants were then discarded and the pellets resuspended in 100 *μ*L of water. The pellets were washed successively in 100 *μ*L of water, and centrifuged twice at 800 ***g*** for 6 min before finally being suspended in a volume of water that made spores easily discernible under the microscope (~2.0 × 10^6^ spores/50 *μ*L).

All aliquots were visualized using a Fluoview FV10i fluorescent microscope (Olympus, Tokyo, Japan). Prepared aliquots were homogenized with a 10 *μ*L pipette and then added to a counting chamber which was then loaded into the FV10i. DAPI and PI filters were selected, and total magnification set to 45×. The FV10i then acquired the map image of the counting chamber, and spores were counted under phase contrast and assigned a live (DAPI only) or dead (DAPI + PI) status. The average number of spores evaluated for viability testing per replicate was 193 ± 12 for water, and 142 ± 5 for 2 M sucrose. Spore viability was assessed using the following calculation:
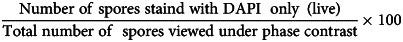


### Infectivity

Frames of eclosing worker brood were collected from non-experimental colonies managed by the Apiculture Program at AAFC Beaverlodge. These frames were taken from colonies determined to be *Nosema* spp.-free *via* PCR as in MacInnis *et al*. (2020). Newly-emerged bees were collected from the frames daily so that all bees being used for infectivity assays were <24 h old, and *Nosema* spp. free.

Infectivity aliquots were prepared as in MacInnis *et al*. (2020). Briefly, a volume containing ~2.2 × 10^6^ spores from each temperature × time combination (per liquid) was centrifuged at 800***g*** for 6 min. After centrifugation, the pellet was resuspended in an appropriate volume of water such that when sugar syrup was added, each bee would receive ~1.0 × 10^5^ spores in 5 *μ*L of 50% (w/w) sucrose.

Newly-emerged bees for the infectivity assay were collected and placed into individual feeding harnesses and starved for 90 min prior to receiving their inoculum. After receiving their 5 *μ*L droplet of inoculum, the newly-emerged bees were given 1 h to consume the droplet. After the hour had passed for consumption, the bees remained in their harnesses for an additional 30 min to prevent any trophallaxis upon caging. Bees that did not consume their inoculum droplets were not caged.

For water, on average, 17 ± 1 bees were successfully inoculated per temperature treatment per timepoint. For 2 M sucrose, the average number of bees successfully inoculated per treatment per timepoint was 21 ± 0.5 bees. Control bees were given a 5 *μ*L droplet of 50% (w/w) sucrose without *N. ceranae*. Once newly-emerged bees were caged, they received 60% (w/w) sucrose solution and a pollen patty containing 25% (by weight) irradiated *B. napus* pollen. The cages were maintained in programmable incubators at 33 ± 1.0°C – for 14 days. After 14 days, all living bees were collected and frozen at −20°C until they could be examined for *N. ceranae* infections. We chose to examine living bees at 14 days post inoculation (dpi) because infections are considered fully developed at this time (Paxton *et al*., 2007; Forsgren and Fries, 2010; Huang and Solter, 2013; Huang *et al*., 2013).

Infectivity was assessed by placing individual bees into 1.5 mL microcentrifuge tubes and adding 1 mL of 70% ethanol to each of the tubes. Each bee was then macerated with a sterile micropestle, and the resulting macerate used to perform a spore count using a Helber Z30000 counting chamber. Spores were enumerated according to Cantwell (1970). Bees were classified as infected if at least two spores were present within the chamber's grid, representing a spore load of 1.5 × 10^5^ spores per bee. Infectivity for each liquid treatment at each time point was calculated using the following equation:
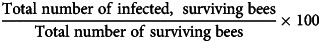


### Statistical analyses

Statistical analyses were performed in ‘R'studio v. 1.1.463 for Mac OS X (R Core Team, 2014). We determined the effect of temperature on *N. ceranae* spore viability in water and 2 M sucrose at six time points. Kruskal–Wallis tests followed by Dunn's test for multiple comparisons (dunn.test, v. 1.3.5., dunn.test) were used to compare the viability of spores in water at all time points (Zar, 2010). A two-way ANOVA was performed on 2 M sucrose and water viability data to determine if there were time × temperature interactions. One-way ANOVAs followed by Tukey's HSD comparisons (TukeyHSD, v. 1.4–13, multcomp) were also used to compare viability in 2 M sucrose at all six time points. All data were assessed for normality using the Shapiro–Wilk test, and by examining qqplots of residuals. Homogeneity of variances was assessed using Bartlett's test for normal data, and Brown Forsythe's test (leveneTest, v.3.0-10, car) for non-normal data (Crawley, 2013).

To estimate 50% viability [when spores are considered to be uninfective (Undeen *et al*., 1993)] for each of the eight temperature × liquid combinations, linear equations were used for seven of the eight treatments, whereas the curve-fitting function of Prism 9 was used for one (−20°C water) treatment (Graphpad, San Diego, CA, USA). To evaluate the appropriateness of linear *vs* non-linear models for each treatment, the curve-fitting function was also applied (Graphpad Software Inc., 2021). After curve-fitting, replicate tests were performed to assess the fit of curves to the non-linear dataset (Draper and Smith, 1998). One-phase decay curves had the lowest discrepancy values (*F* values close to one) and were selected to model the dataset.

Infectivity data (binary-response) were analysed by matrix and time point using Fisher's exact test for equality of proportions followed by multiple pairwise comparisons when necessary (fisher.multcomp, v. 0.9-75, RVAideMemoire), to determine if there was an effect of treatment on infection status (Crawley, 2013). To determine if liquid affected spore intensity (average number of spores in surviving, infected hosts), overall intensity from surviving infected bees from each of the two liquids was compared using Welch's Two Sample *t*-test (t.test, R Stats Package) (Crawley, 2013).

## Results

### Viability

#### Viability in water

**Fig. 1.**
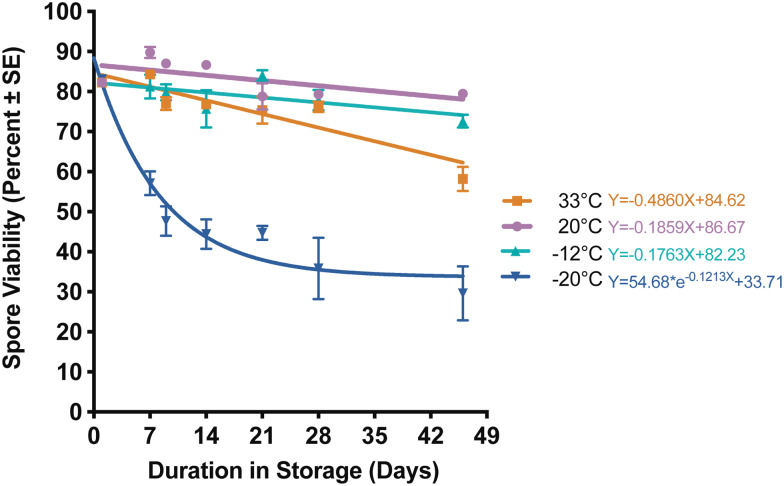
*Nosema ceranae* spore viability for spores stored in water at 33, 20, −12 and −20°C, modelled using linear regression curves. Each point represents mean spore viability (±s.e.; *n* = 3–4 water replicate samples per time point; also refer to Supplementary Table 1 for statistical comparisons).

*Nosema ceranae* spores stored at 33, 20 and −12°C maintained high viability over the course of the experiment. Differences in spore viability were detected as soon as 7 days after storage (*χ*^2^ = 11.65, d.f. = 3, *P* = 0.01) when spore viability at −20°C fell below 60%, however remained above 80% for the remaining three treatments (Fig. 1, Supplemental Table 1). This trend of low viability at −20°C persisted throughout the entire experiment, and is likely what drove the time × temperature interaction (*F*_15,61_ = 2.542, *P* = 0.00536). At five out of six time points, spore viability at −20°C was significantly lower than at 20°C (Fig. 1, Supplemental Table 1). Spores maintained at −20°C reached 50% viability at 6 days (95% CI 3.2–11.3 days) (Table 1), while spores for all three remaining treatments remained above 50% viability at the end of the experiment.

**Table 1.** Fifty per cent viability estimates of *Nosema ceranae* spores in water for up to 46 days or 2 M sucrose for up to 42 days after exposure to 33, 20, −12 and −20°C

Treatment	50% Viability (days)
Water	
33°C	71^a^
20°C	197^a^
−12°C	183^a^
−20°C	6^b^
2 M Sucrose	
33°C	102^a^
20°C	145^a^
−12°C	1439^a^
−20°C	5838^a^

a Estimated using a linear regression equation.

b Estimated using a one-phase decay equation.

#### Viability in 2 M sucrose

**Fig. 2.**
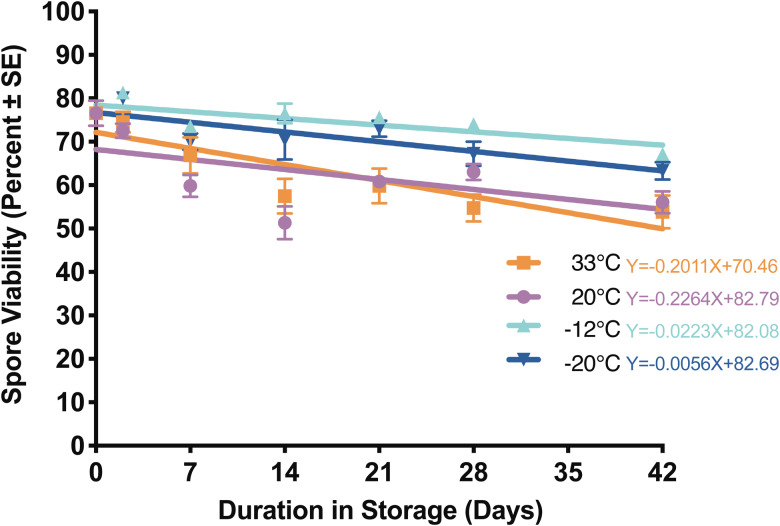
*Nosema ceranae* spore viability for spores stored in 2 M sucrose at 33, 20, −12 and −20°C, modelled using linear regression curves. Each point represents mean spore viability (±s.e.; *n* = 3–5 2 M sucrose replicate samples per time point; also refer to Supplementary Table 2 for statistical comparisons).

*Nosema ceranae* spores stored in 2 M sucrose maintained high viability over the course of the 6-week experiment regardless of storage temperature (Fig. 2, Supplemental Table 2). However, some differences in viability were detected as early as the first timepoint (2 days after storage) when spores stored at 20°C had significantly lower viability than those stored at −12 and −20°C (*F*_3,11_ = 5.26, *P* = 0.01). Interestingly, although there was no time × temperature interaction (*F*_15,75_ = 1.678, *P* = 0.07), spores stored at lower temperatures (−12 and −20°C) maintained higher viability than spores stored at higher temperatures (33 and 20°C) over the course of the experiment (Supplemental Table 2). Spores stored at −12°C experienced <10% decrease in viability over 42 days.

### Infectivity

Mean *N. ceranae* spore intensities for bees used in infectivity experiments were 3.5 × 10^7^ ± 2.4 × 10^6^ spores per bee in water, and 3.4 × 10^7^ ± 1.3 × 10^6^ spores per bee in 2 M sucrose at 14 dpi. There was no difference in mean spore intensity between water and sucrose (*t*_178.72_ = −0.46085, *P* = 0.6455).

#### Infectivity in water

**Fig. 3.**
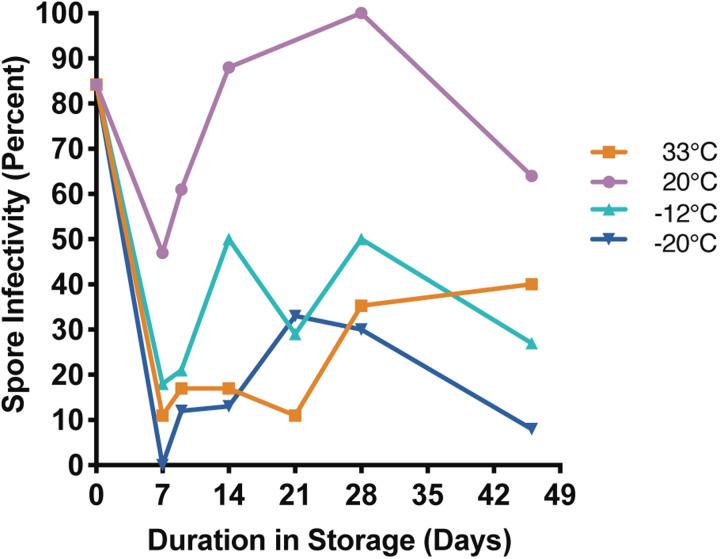
*Nosema ceranae* spore infectivity for spores stored in water at 33, 20, −12 and −20°C for up to 46 days. Each point reflects the percentage of surviving, infected bees at the end of a 14-day incubation period (*n* = 6–19 replicate bees per time point; see Supplemental Table 3 for statistical comparisons).

All treatments experienced a precipitous drop in infectivity after 7 days in storage (Fig. 3, Supplemental Table 3). Despite this drop, and generalized variability over time, all treatment groups did remain infective for the duration of the experiment. Only spores stored at 20°C consistently maintained infectivity above 50% (Fig. 3, Supplemental Table 3). At the last time point (46 days after storage), infectivity for spores stored at −20°C was significantly lower than at 20°C (Supplemental Table 3; *P* = 0.03, d.f. = 3), while viability of this treatment was also lowest out of the four storage temperatures (<30% viable by the end of the experiment) (Fig. 1, Supplemental Table 1).

#### Infectivity in 2 M sucrose

**Fig. 4.**
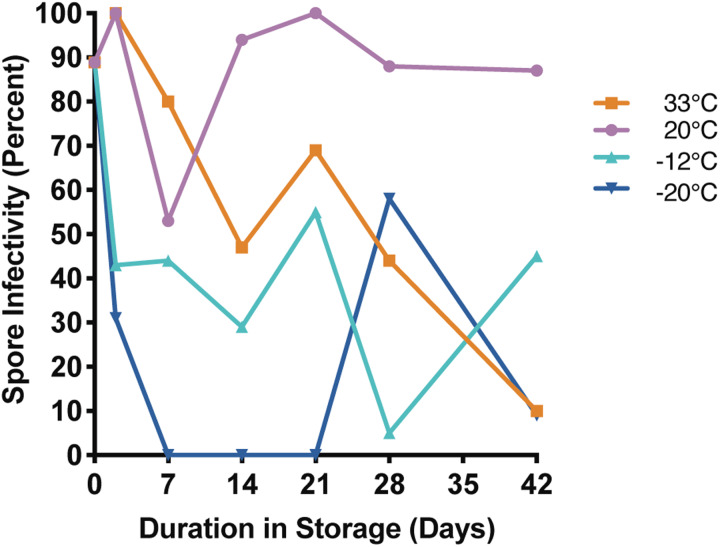
*Nosema ceranae* spore infectivity for spores stored in 2 M sucrose at 33, 20, −12 and −20°C for up to 42 days. Each point reflects the percentage of surviving, infected bees at the end of a 14-day incubation period (*n* = 9–22 replicate bees per time point; see Supplemental Table 4 for statistical comparisons).

*Nosema ceranae* spores kept in 2 M sucrose at 20°C maintained moderate to high infectivity for most of the experiment. At five of the six time points, infectivity was highest for spores belonging to this temperature treatment (Fig. 4, Supplemental Table 4). Infectivity of spores stored at 33°C appeared to decrease with time, and were <50% infective at each of the last two time points (Fig. 4, Supplemental Table 4). Spores stored at −12 and −20°C exhibited highly variable infectivity over the course of the experiment, but were both infective at the last time point. Interestingly, all treatment groups maintained high viability over the course of the experiment, but had much greater variability in infectivity.

## Discussion

This study examines the viability and *in vivo* infectivity of *N. ceranae* spores in liquids associated with honey bees under conditions which are semi-field relevant for northern temperate climates. Storage of *N. ceranae* spores in water at −20°C proved effective at reducing both spore viability and infectivity, while storage at the remaining three temperatures resulted in the maintenance of high viability, and variable infectivity. The low viability and infectivity at −20°C is consistent with previous studies which demonstrate that freezing without a cryoprotectant reduces *N. ceranae* spore survival (Fenoy *et al*., 2009; Fries and Forsgren, 2009; Sánchez Collado *et al*., 2014; McGowan *et al*., 2016). High viability at higher temperatures is also consistent with existing literature (Fenoy *et al*., 2009; Fries and Forsgren, 2009; Martín-Hernández *et al*., 2009; Higes *et al*., 2010; Sánchez Collado *et al*., 2014). Because our study was conducted under semi-field relevant conditions, the data generated provide further insight into the potential survival of the organism with respect to beekeeping practices in northern temperate climates.

Contaminated water (e.g. puddles, water feeders) has previously been reported to act as a source of infective *N. apis* spores for foraging honey bees (Bailey, 1955). This may also be true for *N. ceranae*, especially for contaminated ephemeral water sources available to foraging honey bees during the spring and summer months in temperate climates. *Nosema ceranae* spores stored in water at 20°C in this study maintained high viability and infectivity for the duration of the study (6 weeks). High viability and infectivity over 6 weeks at this temperature suggests that the spores are capable of surviving in ephemeral water sources over the spring and summer months in temperate regions. Because of this high survival, it is likely that *N. ceranae* spores maintained in ephemeral water sources at 20°C are capable of infecting foraging honey bees, as well as other colony members once the foragers return to the hive and exchange water with nestmates.

The high variability in infectivity shown by spores stored in water at all temperature treatments suggests that although contaminated ephemeral water sources may act as a primary route of transmission for *N. ceranae*, spores frozen in water at temperatures warmer than −12°C for a period of time can still be infective. The variability in infectivity may be due to genetic differences in the bees that were infected, or spores that were viable according to fluorescent microscopy were not actually infective. This last point would result in an inflated viability estimate, and further illustrates why we cannot rely on parasite viability estimates to infer infectivity.

Sucrose syrup is typically fed to honey bee colonies during the fall in northern temperate climates to provide them with sufficient carbohydrate stores to survive the winter. Sucrose syrup can be provided to individual colonies in a number of ways including hive top, entrance or internal frame feeders, or may be made accessible to many colonies simultaneously when supplied in large barrels in the centre of an apiary. The use of barrel feeding is common among commercial beekeepers, as this method of feeding is far more efficient than repeatedly applying small amounts of feed to individual colonies. Bees infected with *N. ceranae* can drown in the syrup of a feeding barrel, or can be incidentally crushed by the beekeeper, releasing spores into the syrup and thereby potentially infecting bees from multiple colonies. Additionally, when resources are scarce after nectar flows have ceased in the fall, honey bees will rob weaker colonies of their carbohydrate resources, potentially spreading *N. ceranae* spores.

For *N. ceranae* spores stored in 2 M sucrose, viability was high over the course of the 6-week experiment regardless of storage temperature. Although infectivity was highly variable over the course of the experiment, spores stored at all four temperatures were still infective at the last time point. This high viability combined with the fact that spores remained infective over the course of the experiment, regardless of temperature treatment, suggests that *N. ceranae* could be transmitted *via* 2 M sucrose both within and among colonies with ease. The discrepancy between viability and infectivity measurements in 2 M sucrose (freezing point −3.2°C) again indicates that genetic differences may have existed in the bees that were used for the infectivity experiments, or that spores that appeared viable were not actually infective. Spores appearing as viable but not being infective, especially at the lowest temperature (−20°C) seems possible. Spore membrane integrity would be preserved (accounting for high viability), while other factors, such as the generation of ice crystals within the spores at the low temperatures, might prevent them from germinating effectively. Overall, the high viability and fact that the spores remained infective even at low temperatures at the end of the experiment supports the findings of recent work (McGowan *et al*., 2016; MacInnis *et al*., 2020), providing evidence for cold tolerance, and making the organism's persistence in some temperate climates less conspicuous.

Malone *et al*. (2001) found that spores of *N. apis* had higher viability at 33°C after storage in 50% sucrose rather than honey, which resulted in higher numbers of spores/infected bee. These authors suggest that this result is due to properties of honey such as pH or peroxide activity rather than just osmolarity. Comparing the results of the current study to those of MacInnis *et al*. (2020), a similar trend emerges. Even though spore viability was initially lower for the current study, and although different spore suspensions were used, overall, numbers of spores/bee were higher for spores maintained in 2 M sucrose compared to honey. This further suggests that reduced spore infectivity in honey is due to some property of honey (that is not unique to variety) other than osmolarity. Properties unique to honey could be investigated to potentially provide beekeepers with a novel *Nosema* spp. control.

This study has added much to our knowledge of *N. ceranae* spore biology. It has illustrated that viability cannot always be used to infer infectivity, and it has provided more evidence for cold tolerance.

Based on the results of this study, some economical methods for beekeepers to reduce *N. ceranae* spore loads within their operations without antimicrobials can be provided. When viability is <50%, spores can be considered non-infectious (Undeen *et al*., 1993). Viability for spores maintained in 2 M sucrose did not decrease to half-life, making management recommendations for fall sucrose feed decontamination difficult.

Because 2 M sucrose is a quality resource for honey bees, and is prevalent both within colonies and on equipment, it is probable that 2 M sucrose could act as a primary route of transmission for *N. ceranae* both within and among colonies when the sucrose is present. *Nosema ceranae* spore viability in 2 M sucrose was high among all four temperature treatments for the duration of this experiment, and spores from all four treatments were infective at the last time point. To reduce the potential spread of *N. ceranae* among colonies *via* 2 M sucrose, rather than trying to decontaminate the sucrose, beekeepers could provide colonies with individual sucrose feeders rather than barrel feeding their colonies, and could also use robbing screens or entrance reducers to prevent colonies from robbing one another during the fall dearth when sucrose is being applied.

Due to the low presence of water in/on hive equipment, it seems unlikely that water would act as an important route of transmission for *N. ceranae* within the colony. However, this does not mean that ephemeral water sources in the spring and summer cannot act as sources of spores for *N. ceranae* as both viability and infectivity were high, 79.5 and 64%, respectively, for spores maintained at 20°C at 46 days after storage. Beekeepers concerned about *N. ceranae* transmission *via* water could provide their colonies with water feeders, which is done in some arid regions, and change the water out on a regular basis. Each colony should, preferably, have access to its own feeder, and not a communal feeder to reduce the spread of the parasite between colonies.

We have confirmed the viability and infectivity of *N. ceranae* spores over a range of temperatures in two liquids commonly associated with honey bee colonies. The high viability of *N. ceranae* spores in water at 33, 20 and −12°C, and variable infectivity across all four temperature treatments suggests that although ephemeral water sources may act as a primary route of transmission for *N. ceranae*, spores frozen in water for a period of time can still remain infective to honey bees once thawed. The high viability and variable infectivity of spores in 2 M sucrose indicates that sugar syrup fed to bee colonies during the fall could also act as a primary route of transmission for the parasite within and among colonies. Given the way in which honey bee colonies are managed in northern temperate climates (i.e. fed and overwintered), it seems quite possible that spores could enter the sucrose feed during the feeding process or *via* robbing behaviour, and perpetuate infections in colonies over the winter months when the sucrose is being consumed.

## Data Availability

Supplementary data are available online or from the author on reasonable request.

## Supplementary material

For supplementary material accompanying this paper visit https://doi.org/10.1017/S0031182021001827. click here to view supplementary material
