# Supplementary material for: Honey bees with a drinking problem: potential routes of Nosema ceranae spore transmission
Source: Parasitology. 2021 Nov 4;149(5):573–80. doi: 10.1017/S0031182021001827 (PMC10090605; doi:10.1017/S0031182021001827)
Supplement: Supplementary file 1 [file S0031182021001827sup001.pdf]

Supplemental Table I. Comparisons of *N. ceranae* spore viability in water among temperature treatments across all time points. Kruskal-Wallis tests followed by Dunn's multiple comparisons were used to compare treatments within all six time points. Different letters indicate statistically significant differences among treatments for a given time point ($P<0.05$; Dunn's Test).

Treatment	Viability (Mean % ± SE)	Replicates (n)[α]
**Water Day 7**	χ^2=11.65, df=3, *P*=0.01	
33°C	84.3 ± 0.6**ab**	4
20°C	89.8 ± 1.4**b**	4
-12°C	81.2 ± 3.0**ab**	3
-20°C	57.1 ± 3.0**a**	4
**Water Day 9**	χ^2=11.79, df=3, *P*=0.01	
33°C	77.0 ± 1.6**ab**	4
20°C	87.0 ± 0.7**b**	4
-12°C	79.8 ± 2.0**ab**	4
-20°C	47.7 ± 3.7**a**	3
**Water Day 14**	χ^2=11.03, df=3, *P*=0.01	
33°C	76.8 ± 0.9**ab**	4
20°C	86.7 ± 0.9**b**	4
-12°C	75.7 ± 4.6**ab**	3
-20°C	44.4 ± 3.7**a**	3
**Water Day 21**	χ^2=8.74, df=3, *P*=0.03	
33°C	75.4 ± 3.4**ab**	4
20°C	78.8 ± 3.3**ab**	4
-12°C	84.1 ± 1.3**b**	3
-20°C	44.8 ± 1.8**a**	3
**Water Day 28**	χ^2=8.90, df=3, *P*=0.03	
33°C	76.2 ± 1.3**ab**	4
20°C	79.3 ± 0.7**b**	4
-12°C	77.9 ± 2.5**ab**	3
-20°C	35.8 ± 7.7**a**	3
**Water Day 46**	χ^2=11.27, df=3, *P*=0.01	
33°C	58.2 ± 3.0**ab**	3
20°C	79.5 ± 0.8**b**	4
-12°C	72.6 ± 1.6**ab**	3
-20°C	29.6 ± 6.8**a**	3

[α] Number of spore-containing replicate water samples analyzed per timepoint

Supplemental Table II. Comparisons of *N. ceranae* spore viability in 2M sucrose among temperature treatments across all time points. One-way ANOVA followed by Tukey's HSD was used to compare treatments at all time points. Different letters indicate statistically significant differences among treatments for a given time point ($P<0.05$; Tukey HSD).

Treatment	Viability (Mean % ± SE)	Replicates (n)[α]
**2M Sucrose Day 2**	$F_{3,11}$=5.26, P=0.01	
33°C	74.5 ± 2.3**ab**	4
20°C	72.5 ± 1.7**a**	4
-12°C	81.3 ± 1.4**b**	3
-20°C	80.1 ± 1.5**b**	4
**2M Sucrose Day 7**	$F_{3,13}$=5.11, P=0.01	
33°C	66.9 ± 4.1**ab**	4
20°C	59.9 ± 2.5**a**	5
-12°C	73.6 ± 1.1**b**	4
-20°C	70.0 ± 1.9**ab**	4
**2M Sucrose Day 14**	$F_{3,12}$=9.45, P=0.001	
33°C	57.5 ± 4.0**c**	4
20°C	51.3 ± 3.8**ac**	4
-12°C	76.5 ± 2.3**b**	4
-20°C	70.5 ± 4.6**bc**	4
**2M Sucrose Day 21**	$F_{3,12}$=11.87, P=0.0006	
33°C	59.8 ± 4.0**a**	4
20°C	60.8 ± 1.2**a**	4
-12°C	75.7 ± 1.3**b**	4
-20°C	73.0 ± 1.8**b**	4
**2M Sucrose Day 28**	$F_{3,13}$=11.47, P=0.0005	
33°C	54.8 ± 3.1**a**	4
20°C	63.0 ± 1.8**ac**	5
-12°C	74.1 ± 1.3**b**	4
-20°C	67.2 ± 2.8**bc**	4
**2M Sucrose Day 42**	$F_{3,14}$=5.12, df=3, P=0.01	
33°C	53.9 ± 3.8**a**	5
20°C	56.0 ± 2.6**ab**	5
-12°C	67.2 ± 0.3**b**	4
-20°C	63.3 ± 2.0**ab**	4

[α] Number of spore-containing replicate 2M sucrose samples analyzed per timepoint

Supplemental Table III. Summary of *N. ceranae* spore infectivity in water among all four temperature treatments. Fisher's Exact tests followed by multiple comparisons were used to compare treatments within the six time points. Letters indicate significant differences (P<0.05) between treatments at a given time point.

Treatment	Infectivity (Mean %)	Spore intensity (Mean ± SE)	Replicates (n)[α]
**Water Day 7**	*P*=0.01, df=3		
33°C	11	1.6×10^5	19
20°C	47	$2.0\times10^7 \pm 6.6\times10^6$	19
-12°C	18	$6.2\times10^6 \pm 6.0\times10^6$	11
-20°C	0	0 ± 0	8
**Water Day 9**	*P*=0.006, df=3		
33°C	17**b**	$2.9\times10^7 \pm 2.2\times10^7$	18
20°C	61**a**	$4.6\times10^7 \pm 1.1\times10^7$	18
-12°C	21**b**	$1.1\times10^7 \pm 7.9\times10^6$	19
-20°C	12**b**	$4.5\times10^7 \pm 1.2\times10^7$	17
**Water Day 14**	*P*=0.0001, df=3		
33°C	17**bc**	$7.3\times10^7 \pm 7.6\times10^6$	12
20°C	88**a**	$6.5\times10^7 \pm 6.1\times10^6$	16
-12°C	50**ac**	$2.8\times10^6 \pm 1.8\times10^6$	6
-20°C	13**bc**	5.6×10^6	8
**Water Day 21**	*P*=0.1, df=3		
33°C	11	$8.3\times10^6 \pm 8.1\times10^6$	19
20°C	-	-	-
-12°C	29	$5.7\times10^7 \pm 9.1\times10^6$	17
-20°C	33	$2.7\times10^7 \pm 5.6\times10^6$	12
**Water Day 28**	*P*=0.0001, df=3		
33°C	35**b**	$3.1\times10^7 \pm 8.0\times10^6$	17
20°C	100**a**	$3.6\times10^7 \pm 4.3\times10^6$	15
-12°C	50**b**	$3.2\times10^7 \pm 4.6\times10^6$	14
-20°C	30**b**	$3.1\times10^7 \pm 4.8\times10^6$	10
**Water Day 46**	*P*=0.03 df=3		
33°C	40**ab**	$2.4\times10^7 \pm 6.8\times10^6$	15
20°C	64**a**	$3.7\times10^7 \pm 6.7\times10^6$	11
-12°C	27**ab**	$3.8\times10^7 \pm 9.3\times10^6$	11
-20°C	8**b**	1.6×10^7	13

[α] Number of surviving bees inoculated with water-treated spores at the end of a 14-day incubation period

Supplemental Table IV. Summary of *N. ceranae* spore infectivity in 2M sucrose among all four temperature treatments. Fisher's Exact tests followed by multiple comparisons were used to compare treatments within the six time points. Letters indicate significant differences (P<0.05) between treatments at a given time point.

Treatment	Infectivity (Mean %)	Spore intensity (Mean ± SE)	Replicates (n)[α]
**2M Sucrose Day 2**	P=2.5×10^{-6}, df=3		
33°C	100**a**	2.8×10^{7} ± 3.8×10^{6}	16
20°C	100**a**	4.3×10^{7} ± 4.6×10^{6}	12
-12°C	43**b**	2.1×10^{7} ± 4.6×10^{6}	21
-20°C	31**b**	1.7×10^{7} ± 5.9×10^{6}	13
**2M Sucrose Day 7**	P=1.9×10^{-5}, df=3		
33°C	80**a**	3.3×10^{7} ± 5.7×10^{6}	15
20°C	53**b**	3.9×10^{7} ± 7.2×10^{6}	15
-12°C	44**b**	3.1×10^{7} ± 5.8×10^{6}	18
-20°C	0**c**	0	16
**2M Sucrose Day 14**	P=4.1×10^{-8}, df=3		
33°C	47**b**	4.4×10^{7} ± 5.2×10^{6}	15
20°C	94**a**	3.5×10^{7} ± 3.5×10^{6}	16
-12°C	29**b**	5.8×10^{7} ± 6.8×10^{6}	14
-20°C	0**c**	0	17
**2M Sucrose Day 21**	P=2.4×10^{-6}, df=3		
33°C	69**a**	4.2×10^{7} ± 8.1×10^{6}	16
20°C	100**a**	4.5×10^{7} ± 3.7×10^{6}	15
-12°C	55**ab**	4.1×10^{7} ± 3.8×10^{6}	20
-20°C	0**bc**	0	9
**2M Sucrose Day 28**	P=2.5×10^{-8}, df=3		
33°C	44**b**	2.7×10^{7} ± 7.7×10^{6}	18
20°C	88**a**	3.5×10^{7} ± 3.4×10^{6}	16
-12°C	5**c**	2.8×10^{7}	20
-20°C	58**b**	1.2×10^{7} ± 3.8×10^{6}	19
**2M Sucrose Day 42**	P=1.6×10^{-11} df=3		
33°C	10**c**	2.3×10^{7} ± 2.3×10^{7}	20
20°C	87**a**	2.9×10^{7} ± 3.7×10^{6}	15
-12°C	45**b**	3.6×10^{7} ± 4.6×10^{6}	20
-20°C	9**c**	4.4×10^{6} ± 4.2×10^{6}	22

[α] Number of surviving bees inoculated with 2M sucrose-treated spores at the end of a 14-day incubation period
